# (2*R*)-*N*-[5-(4-Chloro­phen­yl)-1,3,4-thia­diazol-2-yl]-2-(cinnamoylamino)propanamide

**DOI:** 10.1107/S1600536808030353

**Published:** 2008-09-24

**Authors:** Shao-Hua Li, Hui-Ming Huang, Bin-Hai Kuang, Guo-Gang Tu, Cheng-Mei Liu

**Affiliations:** a State Key Laboratory of Food Science and Technology, Nanchang University, 330047 Nanchang, JiangXi, People’s Republic of China; b Department of Pharmacy, NanChang University Medical College, 330006 Nanchang, JiangXi, People’s Republic of China

## Abstract

In the title compound, C_20_H_17_ClN_4_O_2_S, the dihedral angle between the two benzene rings is 65.9 (1)°; the corresponding angle between the 4-chloro­phenyl and thia­diazole rings is 3.4 (8)°. The conformations of the N—H and C=O bonds are *anti* with respect to each other. The enone groups show a *trans* configuration. The structure displays intermolecular N—H⋯O, C—H⋯N, C—H⋯S and C—H⋯O hydrogen bonding.

## Related literature

For 1,3,4-thia­diazole scaffold compounds and their biological activity, see: Tu *et al.* (2008 ▶). For the synthesis, see: Foroumadi *et al.* (1999 ▶); Levy & Palmer (1942 ▶); Song *et al.* (1992 ▶). For related structures, see: Fun *et al.* (2008 ▶); Gowda *et al.* (2008 ▶) Thiruvalluvar *et al.* (2008 ▶).
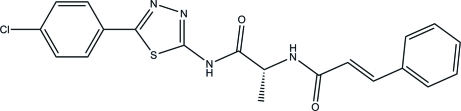


## Experimental

### 

#### Crystal data

C_20_H_17_ClN_4_O_2_S
*M* *_r_* = 412.89
Orthorhombic, 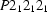

*a* = 6.6324 (15) Å
*b* = 8.575 (2) Å
*c* = 34.367 (8) Å
*V* = 1954.6 (8) Å^3^
*Z* = 4
Mo *K*α radiation
μ = 0.33 mm^−1^
*T* = 296 (2) K
0.41 × 0.17 × 0.07 mm

#### Data collection

Bruker APEXII CCD area-detector diffractometer
Absorption correction: none
14721 measured reflections
4706 independent reflections
2807 reflections with *I* > 2σ(*I*)
*R*_int_ = 0.046

#### Refinement

*R*[*F* ^2^ > 2σ(*F* ^2^)] = 0.043
*wR*(*F* ^2^) = 0.099
*S* = 1.02
4706 reflections
254 parameters
H-atom parameters constrained
Δρ_max_ = 0.15 e Å^−3^
Δρ_min_ = −0.20 e Å^−3^
Absolute structure: Flack (1983 ▶), 1876 Friedel pairs
Flack parameter: −0.12 (7)

### 

Data collection: *APEX2* (Bruker, 2004 ▶); cell refinement: *SAINT* (Bruker, 2004 ▶); data reduction: *SAINT*; program(s) used to solve structure: *SHELXS97* (Sheldrick, 2008 ▶); program(s) used to refine structure: *SHELXL97* (Sheldrick, 2008 ▶); molecular graphics: *APEX2*; software used to prepare material for publication: *APEX2* and *publCIF* (Westrip, 2008 ▶).

## Supplementary Material

Crystal structure: contains datablocks I, global. DOI: 10.1107/S1600536808030353/gw2052sup1.cif

Structure factors: contains datablocks I. DOI: 10.1107/S1600536808030353/gw2052Isup2.hkl

Additional supplementary materials:  crystallographic information; 3D view; checkCIF report

## Figures and Tables

**Table 1:** Hydrogen-bond geometry (Å, °)

*D*—H⋯*A*	*D*—H	H⋯*A*	*D*⋯*A*	*D*—H⋯*A*
N2—H2*A*⋯O1^i^	0.86	1.94	2.802 (3)	175
C7—H7*A*⋯N3^ii^	0.93	2.54	3.446 (3)	164
C11—H11*C*⋯S1^iii^	0.96	2.77	3.526 (3)	136
C20—H20*A*⋯O2^iv^	0.93	2.48	3.380 (3)	162

Symmetry codes: (i) 
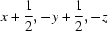
; (ii) 
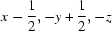
; (iii) 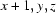; (iv) 
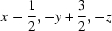
.

**Fig. 1.**
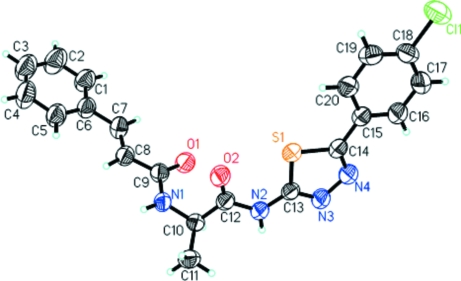
Molecular structure of the title compound. Displacement ellipsoids are drawn at the 50% probability level.

**Fig. 2.**
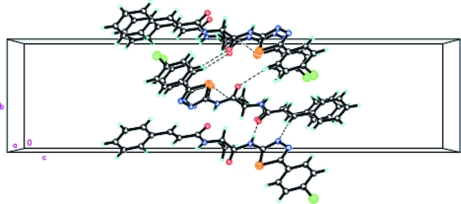
The crystal packing of title compound, viewed along the a axis with hydrogen bonds drawn as dashed lines.

## supplementary crystallographic information

### Comment

In our previous work, 1,3,4-thiadiazole scaffold compounds and their biological activity have been studied (Tu *et al.*, 2008). In view of the importance of these organic materials, the title compound (Fig. 1) was synthesized (Foroumadi *et al.*, 1999; Levy & Palmer 1942; Song *et al.*, 1992) and its crystal structure is reported here. The structure of title compound, C_20_H_17_ClN_4_O_2_S, has orthorhombic (*P*2_1_2_1_2_1_) symmetry. The dihedral angles between the *p*-cholobenzene and thiadiazol rings is 3.4 (8) °, the corresponding values between the two benzene rings are measured to 65.9 (1)°. The conformations of the N—H and C=O bonds are anti with respect to each other. The enone groups are *trans* configurated. Bond lengths and angles are in normal ranges and comparable to those in related structures (Gowda *et al.*, 2008; Fun *et al.*, 2008; Thiruvalluvar *et al.*, 2008). In the crystal structure, molecules are linked through intermolecular hydrogen bonds forming a three-dimensional network (Table 1, Figure 2).

### Experimental

*N,N*-Dicyclohexylcarbodiimide (5.7 mmol) was added to a cooled solution of *N*-cinnamoyl-*D*-alanine (5.6 mmol) and *N*-hydroxysuccinimide (5.6 mmol) in freshly distillation dioxane (30 ml). The reaction mixture was stirred overnight at room temperature. The insoluble material was filtered off and washed with cold dioxane. 2-Amino-5-(4-choloxyphenyl)-1,3,4-thiadiazole (5.5 mmol) was added to the filtrate and the reaction mixture was stirred for 48 hr at room temperature. The solvent was removed under reduced pressure. The residual was dissolved in EtOAc and the insoluble material was filtered off. The filtrate was washed successively with saturated Na_2_CO_3_ solution(20 ml, *x* 3), water(20 ml, *x* 1), 0.1 *M* HCl(20 ml, *x* 3) and water(20 ml, *x* 1). The organic layer evaporated *in vacuo*, the residual was recrystallized from methanol. Colorless block-shaped single crystals of the title compound suitable for X-ray diffraction analysis precipitated after several days.

### Refinement

H atoms were positioned geometrically and refined using a riding model using *SHELXL97* default values (*U*_ĩso_(H) = 1.2 *U*_eq_(C) for CH and CH_2_ groups and U_ĩso_(H) = 1.5 *U*_eq_(C) for CH_3_). Refinement with all data (Friedel opposites not merged) led to an unsuitably large error of the Flack parameter. The final refinement was therefore performed with a data set with merged Friedel pairs, hence the calculated Flack parameter is meaningless. The absolute configuration is nevertheless undoubtly as described since enantiomerically pure starting compounds were used and the reaction conditions are not condidered to lead to racemization or inversion.

### Crystal data

**Table:**

C_20_H_17_ClN_4_O_2_S	*D*_x_ = 1.403 Mg m^−^^3^
*M**_r_* = 412.89	Melting point: 480 K
Orthorhombic, *P*2_1_2_1_2_1_	Mo *K*α radiation, λ = 0.71073 Å
Hall symbol: P 2ac 2ab	Cell parameters from 3308 reflections
*a* = 6.6324 (15) Å	θ = 2.4–21.0°
*b* = 8.575 (2) Å	µ = 0.33 mm^−^^1^
*c* = 34.367 (8) Å	*T* = 296 K
*V* = 1954.6 (8) Å^3^	Bolck, colourless
*Z* = 4	0.41 × 0.18 × 0.07 mm
*F*(000) = 856	

### Data collection

**Table:**

Bruker SMART CCD area-detector diffractometer	2807 reflections with *I* > 2σ(*I*)
Radiation source: fine-focus sealed tube	*R*_int_ = 0.046
graphite	θ_max_ = 28.4°, θ_min_ = 2.4°
φ and ω scans	*h* = −8→8
14721 measured reflections	*k* = −11→11
4706 independent reflections	*l* = −45→44

### Refinement

**Table:**

Refinement on *F*^2^	Secondary atom site location: difference Fourier map
Least-squares matrix: full	Hydrogen site location: inferred from neighbouring sites
*R*[*F*^2^ > 2σ(*F*^2^)] = 0.043	H-atom parameters constrained
*wR*(*F*^2^) = 0.099	*w* = 1/[σ^2^(*F*_o_^2^) + (0.0226*P*)^2^ + 0.0747*P*] where *P* = (*F*_o_^2^ + 2*F*_c_^2^)/3
*S* = 1.02	(Δ/σ)_max_ < 0.001
4706 reflections	Δρ_max_ = 0.15 e Å^−^^3^
254 parameters	Δρ_min_ = −0.20 e Å^−^^3^
0 restraints	Absolute structure: Flack (1983), 1876 Friedel pairs
Primary atom site location: structure-invariant direct methods	Flack parameter: −0.12 (7)

### Special details

**Table:**

Geometry. All e.s.d.'s (except the e.s.d. in the dihedral angle between two l.s. planes) are estimated using the full covariance matrix. The cell e.s.d.'s are taken into account individually in the estimation of e.s.d.'s in distances, angles and torsion angles; correlations between e.s.d.'s in cell parameters are only used when they are defined by crystal symmetry. An approximate (isotropic) treatment of cell e.s.d.'s is used for estimating e.s.d.'s involving l.s. planes.
Refinement. Refinement of *F*^2^ against ALL reflections. The weighted *R*-factor *wR* and goodness of fit *S* are based on *F*^2^, conventional *R*-factors *R* are based on *F*, with *F* set to zero for negative *F*^2^. The threshold expression of *F*^2^ > σ(*F*^2^) is used only for calculating *R*-factors(gt) *etc*. and is not relevant to the choice of reflections for refinement. *R*-factors based on *F*^2^ are statistically about twice as large as those based on *F*, and *R*- factors based on ALL data will be even larger.

### Fractional atomic coordinates and isotropic or equivalent isotropic displacement parameters (Å^2^)

**Table:**

	*x*	*y*	*z*	*U*_iso_*/*U*_eq_	
C1	0.6789 (5)	0.2710 (4)	0.19355 (8)	0.0782 (9)	
H1B	0.6067	0.2030	0.1777	0.094*	
C2	0.6159 (6)	0.2979 (4)	0.23129 (10)	0.0996 (13)	
H2B	0.5024	0.2468	0.2408	0.120*	
C3	0.7184 (6)	0.3981 (5)	0.25465 (9)	0.0977 (12)	
H3B	0.6745	0.4158	0.2800	0.117*	
C4	0.8862 (6)	0.4732 (4)	0.24098 (8)	0.0937 (12)	
H4B	0.9551	0.5434	0.2568	0.112*	
C5	0.9526 (5)	0.4443 (4)	0.20360 (8)	0.0756 (9)	
H5A	1.0692	0.4927	0.1947	0.091*	
C6	0.8481 (5)	0.3443 (3)	0.17924 (7)	0.0613 (8)	
C7	0.9087 (5)	0.3172 (3)	0.13882 (7)	0.0612 (8)	
H7A	0.8246	0.2533	0.1242	0.073*	
C8	1.0669 (4)	0.3722 (3)	0.12100 (7)	0.0584 (7)	
H8A	1.1579	0.4314	0.1353	0.070*	
C9	1.1094 (4)	0.3464 (3)	0.07984 (7)	0.0523 (7)	
C10	1.3357 (4)	0.4044 (3)	0.02573 (6)	0.0483 (6)	
H10A	1.3421	0.2934	0.0190	0.058*	
C11	1.5445 (4)	0.4755 (3)	0.02035 (8)	0.0626 (8)	
H11A	1.6384	0.4245	0.0374	0.094*	
H11B	1.5397	0.5847	0.0264	0.094*	
H11C	1.5870	0.4619	−0.0061	0.094*	
C12	1.1836 (4)	0.4831 (3)	−0.00072 (7)	0.0494 (6)	
C13	1.0385 (4)	0.4721 (3)	−0.06496 (7)	0.0471 (6)	
C14	0.7573 (4)	0.5583 (3)	−0.10172 (6)	0.0494 (6)	
C15	0.5693 (4)	0.6256 (3)	−0.11652 (7)	0.0490 (6)	
C16	0.4931 (5)	0.5842 (3)	−0.15249 (7)	0.0639 (7)	
H16A	0.5625	0.5115	−0.1675	0.077*	
C17	0.3163 (5)	0.6479 (3)	−0.16687 (8)	0.0669 (8)	
H17A	0.2679	0.6198	−0.1913	0.080*	
C18	0.2142 (4)	0.7538 (3)	−0.14421 (8)	0.0589 (7)	
C19	0.2848 (5)	0.7959 (3)	−0.10843 (8)	0.0637 (8)	
H19A	0.2137	0.8676	−0.0935	0.076*	
C20	0.4600 (4)	0.7328 (3)	−0.09455 (8)	0.0591 (7)	
H20A	0.5068	0.7619	−0.0701	0.071*	
Cl1	−0.00738 (13)	0.83679 (9)	−0.16181 (2)	0.0835 (3)	
N1	1.2732 (3)	0.4188 (2)	0.06588 (5)	0.0537 (5)	
H1A	1.3436	0.4758	0.0813	0.064*	
N2	1.1824 (3)	0.4305 (2)	−0.03825 (5)	0.0525 (5)	
H2A	1.2768	0.3681	−0.0455	0.063*	
N3	1.0265 (4)	0.4021 (3)	−0.09856 (6)	0.0590 (6)	
N4	0.8619 (4)	0.4535 (2)	−0.12001 (6)	0.0585 (6)	
O1	1.0027 (3)	0.2679 (2)	0.05768 (5)	0.0672 (5)	
O2	1.0712 (3)	0.5869 (2)	0.00979 (5)	0.0638 (5)	
S1	0.85258 (10)	0.60793 (7)	−0.056449 (17)	0.05201 (18)	

### Atomic displacement parameters (Å^2^)

**Table:**

	*U*^11^	*U*^22^	*U*^33^	*U*^12^	*U*^13^	*U*^23^
C1	0.081 (3)	0.090 (2)	0.0635 (18)	−0.017 (2)	0.0140 (18)	−0.0047 (16)
C2	0.112 (4)	0.116 (3)	0.070 (2)	−0.030 (3)	0.029 (2)	−0.004 (2)
C3	0.110 (3)	0.124 (3)	0.0594 (19)	−0.021 (3)	0.021 (2)	−0.006 (2)
C4	0.113 (3)	0.114 (3)	0.0541 (18)	−0.028 (3)	0.0073 (19)	−0.0090 (18)
C5	0.080 (2)	0.093 (2)	0.0539 (17)	−0.0173 (19)	0.0071 (16)	−0.0014 (15)
C6	0.068 (2)	0.0673 (18)	0.0484 (14)	−0.0030 (16)	0.0023 (14)	0.0034 (12)
C7	0.075 (2)	0.0623 (16)	0.0465 (15)	−0.0090 (16)	−0.0007 (15)	0.0002 (13)
C8	0.0591 (19)	0.0670 (17)	0.0490 (15)	−0.0077 (15)	0.0026 (13)	−0.0025 (13)
C9	0.0530 (19)	0.0535 (15)	0.0503 (14)	−0.0074 (13)	−0.0006 (13)	0.0012 (12)
C10	0.0448 (15)	0.0539 (14)	0.0464 (13)	−0.0036 (14)	0.0008 (12)	−0.0019 (11)
C11	0.0491 (19)	0.0728 (18)	0.0658 (17)	−0.0078 (15)	0.0092 (14)	−0.0079 (14)
C12	0.0496 (18)	0.0524 (15)	0.0460 (14)	−0.0044 (13)	0.0050 (12)	−0.0053 (11)
C13	0.0461 (16)	0.0516 (13)	0.0437 (13)	0.0004 (12)	0.0065 (12)	−0.0024 (11)
C14	0.0524 (18)	0.0529 (15)	0.0428 (13)	−0.0032 (13)	0.0074 (12)	−0.0003 (11)
C15	0.0502 (17)	0.0516 (14)	0.0453 (14)	−0.0025 (13)	0.0051 (12)	0.0000 (12)
C16	0.067 (2)	0.0747 (18)	0.0499 (15)	0.0125 (17)	0.0012 (15)	−0.0069 (13)
C17	0.065 (2)	0.081 (2)	0.0540 (15)	0.0083 (17)	−0.0075 (15)	−0.0055 (14)
C18	0.0460 (18)	0.0622 (17)	0.0686 (18)	0.0037 (14)	−0.0010 (14)	0.0109 (14)
C19	0.055 (2)	0.0669 (18)	0.0695 (18)	0.0050 (15)	0.0080 (15)	−0.0077 (14)
C20	0.056 (2)	0.0666 (17)	0.0545 (16)	0.0020 (16)	0.0009 (14)	−0.0103 (13)
Cl1	0.0582 (5)	0.0923 (6)	0.1001 (6)	0.0079 (4)	−0.0089 (5)	0.0129 (4)
N1	0.0521 (14)	0.0672 (13)	0.0417 (11)	−0.0115 (12)	−0.0006 (10)	−0.0046 (10)
N2	0.0508 (14)	0.0629 (13)	0.0439 (11)	0.0086 (11)	0.0030 (10)	−0.0048 (9)
N3	0.0611 (16)	0.0685 (13)	0.0472 (12)	0.0108 (13)	0.0006 (11)	−0.0074 (11)
N4	0.0591 (16)	0.0676 (13)	0.0488 (11)	0.0069 (13)	0.0014 (12)	−0.0090 (10)
O1	0.0605 (13)	0.0815 (12)	0.0595 (11)	−0.0230 (11)	0.0052 (11)	−0.0127 (10)
O2	0.0666 (13)	0.0715 (12)	0.0534 (10)	0.0174 (11)	−0.0024 (9)	−0.0166 (9)
S1	0.0507 (4)	0.0584 (4)	0.0469 (3)	0.0047 (3)	0.0040 (3)	−0.0059 (3)

### Geometric parameters (Å, °)

**Table:**

C1—C6	1.377 (4)	C11—H11C	0.9600
C1—C2	1.382 (4)	C12—O2	1.216 (3)
C1—H1B	0.9300	C12—N2	1.367 (3)
C2—C3	1.359 (5)	C13—N3	1.304 (3)
C2—H2B	0.9300	C13—N2	1.372 (3)
C3—C4	1.369 (5)	C13—S1	1.721 (3)
C3—H3B	0.9300	C14—N4	1.298 (3)
C4—C5	1.380 (4)	C14—C15	1.465 (3)
C4—H4B	0.9300	C14—S1	1.732 (2)
C5—C6	1.384 (4)	C15—C16	1.382 (3)
C5—H5A	0.9300	C15—C20	1.393 (3)
C6—C7	1.465 (4)	C16—C17	1.385 (4)
C7—C8	1.303 (4)	C16—H16A	0.9300
C7—H7A	0.9300	C17—C18	1.375 (4)
C8—C9	1.459 (3)	C17—H17A	0.9300
C8—H8A	0.9300	C18—C19	1.364 (4)
C9—O1	1.239 (3)	C18—Cl1	1.741 (3)
C9—N1	1.340 (3)	C19—C20	1.368 (4)
C10—N1	1.446 (3)	C19—H19A	0.9300
C10—C12	1.517 (3)	C20—H20A	0.9300
C10—C11	1.524 (3)	N1—H1A	0.8600
C10—H10A	0.9800	N2—H2A	0.8600
C11—H11A	0.9600	N3—N4	1.389 (3)
C11—H11B	0.9600		
			
C6—C1—C2	120.4 (3)	H11B—C11—H11C	109.5
C6—C1—H1B	119.8	O2—C12—N2	121.2 (2)
C2—C1—H1B	119.8	O2—C12—C10	123.8 (2)
C3—C2—C1	120.6 (4)	N2—C12—C10	115.0 (2)
C3—C2—H2B	119.7	N3—C13—N2	121.0 (2)
C1—C2—H2B	119.7	N3—C13—S1	114.7 (2)
C2—C3—C4	120.1 (3)	N2—C13—S1	124.11 (17)
C2—C3—H3B	120.0	N4—C14—C15	124.0 (2)
C4—C3—H3B	120.0	N4—C14—S1	114.2 (2)
C3—C4—C5	119.6 (3)	C15—C14—S1	121.72 (18)
C3—C4—H4B	120.2	C16—C15—C20	117.6 (2)
C5—C4—H4B	120.2	C16—C15—C14	121.4 (2)
C4—C5—C6	121.0 (3)	C20—C15—C14	121.0 (2)
C4—C5—H5A	119.5	C15—C16—C17	121.8 (3)
C6—C5—H5A	119.5	C15—C16—H16A	119.1
C1—C6—C5	118.3 (3)	C17—C16—H16A	119.1
C1—C6—C7	119.3 (3)	C18—C17—C16	118.4 (3)
C5—C6—C7	122.3 (3)	C18—C17—H17A	120.8
C8—C7—C6	127.5 (3)	C16—C17—H17A	120.8
C8—C7—H7A	116.2	C19—C18—C17	121.1 (3)
C6—C7—H7A	116.2	C19—C18—Cl1	119.6 (2)
C7—C8—C9	123.8 (3)	C17—C18—Cl1	119.3 (2)
C7—C8—H8A	118.1	C18—C19—C20	120.1 (3)
C9—C8—H8A	118.1	C18—C19—H19A	120.0
O1—C9—N1	119.7 (2)	C20—C19—H19A	120.0
O1—C9—C8	124.6 (3)	C19—C20—C15	121.0 (3)
N1—C9—C8	115.7 (2)	C19—C20—H20A	119.5
N1—C10—C12	110.1 (2)	C15—C20—H20A	119.5
N1—C10—C11	110.0 (2)	C9—N1—C10	122.3 (2)
C12—C10—C11	110.7 (2)	C9—N1—H1A	118.8
N1—C10—H10A	108.7	C10—N1—H1A	118.8
C12—C10—H10A	108.7	C12—N2—C13	123.3 (2)
C11—C10—H10A	108.7	C12—N2—H2A	118.3
C10—C11—H11A	109.5	C13—N2—H2A	118.3
C10—C11—H11B	109.5	C13—N3—N4	111.8 (2)
H11A—C11—H11B	109.5	C14—N4—N3	112.5 (2)
C10—C11—H11C	109.5	C13—S1—C14	86.70 (12)
H11A—C11—H11C	109.5		
			
C6—C1—C2—C3	0.7 (6)	C16—C17—C18—C19	0.2 (4)
C1—C2—C3—C4	−0.3 (6)	C16—C17—C18—Cl1	179.5 (2)
C2—C3—C4—C5	−1.2 (6)	C17—C18—C19—C20	0.1 (4)
C3—C4—C5—C6	2.2 (5)	Cl1—C18—C19—C20	−179.2 (2)
C2—C1—C6—C5	0.2 (5)	C18—C19—C20—C15	0.2 (4)
C2—C1—C6—C7	−178.4 (3)	C16—C15—C20—C19	−0.8 (4)
C4—C5—C6—C1	−1.7 (5)	C14—C15—C20—C19	179.8 (2)
C4—C5—C6—C7	176.8 (3)	O1—C9—N1—C10	−2.3 (4)
C1—C6—C7—C8	−177.5 (3)	C8—C9—N1—C10	179.8 (2)
C5—C6—C7—C8	4.0 (5)	C12—C10—N1—C9	67.6 (3)
C6—C7—C8—C9	−176.3 (2)	C11—C10—N1—C9	−170.1 (2)
C7—C8—C9—O1	−1.3 (4)	O2—C12—N2—C13	−9.9 (4)
C7—C8—C9—N1	176.5 (3)	C10—C12—N2—C13	171.0 (2)
N1—C10—C12—O2	24.2 (3)	N3—C13—N2—C12	−170.0 (2)
C11—C10—C12—O2	−97.7 (3)	S1—C13—N2—C12	5.7 (3)
N1—C10—C12—N2	−156.8 (2)	N2—C13—N3—N4	175.5 (2)
C11—C10—C12—N2	81.4 (3)	S1—C13—N3—N4	−0.5 (3)
N4—C14—C15—C16	−2.3 (4)	C15—C14—N4—N3	−176.1 (2)
S1—C14—C15—C16	−179.3 (2)	S1—C14—N4—N3	1.1 (3)
N4—C14—C15—C20	177.1 (2)	C13—N3—N4—C14	−0.4 (3)
S1—C14—C15—C20	0.1 (3)	N3—C13—S1—C14	0.9 (2)
C20—C15—C16—C17	1.1 (4)	N2—C13—S1—C14	−175.0 (2)
C14—C15—C16—C17	−179.5 (2)	N4—C14—S1—C13	−1.2 (2)
C15—C16—C17—C18	−0.8 (4)	C15—C14—S1—C13	176.1 (2)

### Hydrogen-bond geometry (Å, °)

**Table:**

*D*—H···*A*	*D*—H	H···*A*	*D*···*A*	*D*—H···*A*
N2—H2A···O1^i^	0.86	1.94	2.802 (3)	175.
C7—H7A···O1	0.93	2.58	2.888 (3)	100.
C7—H7A···N3^ii^	0.93	2.54	3.446 (3)	164.
C11—H11C···S1^iii^	0.96	2.77	3.526 (3)	136.
C20—H20A···S1	0.93	2.69	3.105 (3)	108.
C20—H20A···O2^iv^	0.93	2.48	3.380 (3)	162.

Symmetry codes: (i) *x*+1/2, −*y*+1/2, −*z*; (ii) *x*−1/2, −*y*+1/2, −*z*; (iii) *x*+1, *y*, *z*; (iv) *x*−1/2, −*y*+3/2, −*z*.
